# Supplementary material for: Mechanisms governing the pioneering and redistribution capabilities of the non-classical pioneer PU.1
Source: Nat Commun. 2020 Jan 21;11:402. doi: 10.1038/s41467-019-13960-2 (PMC6972792; doi:10.1038/s41467-019-13960-2)
Supplement: Supplementary file 7 — Source data [file 41467_2019_13960_MOESM7_ESM.zip › Source_Data/FigureS2/FigureS2B_MotifScanOutput/KmeansCluster9/homerResults/motif1.similar.html]

motif1

## Information for motif1

A
T
G
C
C
T
G
A
A
T
G
C
C
G
A
T
A
C
G
T
A
G
T
C
A
G
T
C
A
G
C
T
A
G
T
C
G
C
A
T
G
A
T
C
G
C
A
T
  
Reverse Opposite:  

C
G
T
A
C
T
A
G
C
G
T
A
T
C
A
G
T
C
G
A
C
T
A
G
A
C
T
G
C
G
T
A
C
G
T
A
T
A
C
G
G
A
C
T
T
A
C
G
  

|  |  |
| --- | --- |
| p-value: | 1e-1190 |
| log p-value: | -2.741e+03 |
| Information Content per bp: | 1.650 |
| Number of Target Sequences with motif | 1848.0 |
| Percentage of Target Sequences with motif | 81.20% |
| Number of Background Sequences with motif | 5220.5 |
| Percentage of Background Sequences with motif | 11.31% |
| Average Position of motif in Targets | 102.1 +/- 26.2bp |
| Average Position of motif in Background | 101.2 +/- 71.1bp |
| Strand Bias (log2 ratio + to - strand density) | 0.0 |
| Multiplicity (# of sites on avg that occur together) | 1.16 |
| Motif File: | file (matrix) reverse opposite |

### Similar de novo motifs found

|  |  |  |  |  |  |  |  |
| --- | --- | --- | --- | --- | --- | --- | --- |
| Rank | Match Score | Redundant Motif | P-value | log P-value | % of Targets | % of Background | Motif file |
| 1 | 0.977 | A T G C C T G A A T G C G A C T C G A T T G A C G A T C A G C T A G T C G C A T G A C T | 1e-1151 | -2651.475349 | 73.59% | 8.23% | motif file (matrix) |
| 2 | 0.988 | A G T C A T G C C G T A A T G C A C G T A C G T A G T C A G T C A G C T A G T C G C A T G A C T G A C T | 1e-1079 | -2485.330084 | 64.85% | 5.77% | motif file (matrix) |
| 3 | 0.978 | C T G A T C A G T C G A A C T G C T A G C G T A G C T A T A C G G A C T T A C G | 1e-1075 | -2476.909125 | 79.61% | 12.50% | motif file (matrix) |
| 4 | 0.965 | C A T G C T G A C T G A C T G A T C A G C T G A A C T G A C T G C G T A C G T A C T A G A C G T A T C G T A G C | 1e-1013 | -2333.629995 | 70.21% | 8.80% | motif file (matrix) |
| 5 | 0.956 | T A C G T C G A A C T G A C T G C G T A C G T A T C A G G C A T T A C G | 1e-1004 | -2313.432739 | 66.12% | 7.20% | motif file (matrix) |
| 6 | 0.948 | A T G C C G T A A G T C A C G T A C G T A G T C A G T C A G C T | 1e-885 | -2039.205694 | 56.28% | 5.18% | motif file (matrix) |
| 7 | 0.909 | A G T C C G T A A G T C A C G T A C G T A G T C A G T C | 1e-737 | -1698.071749 | 51.36% | 5.43% | motif file (matrix) |
| 8 | 0.941 | T A C G T C G A A T C G A C T G G T C A T C G A T A C G G A C T | 1e-690 | -1589.481847 | 90.69% | 33.05% | motif file (matrix) |
| 9 | 0.935 | C T G A A T G C A C G T A G C T A G T C A G T C A G C T A G T C G A C T | 1e-665 | -1533.206865 | 92.18% | 35.93% | motif file (matrix) |
| 10 | 0.909 | A T G C C T G A A G T C G C A T C A G T T A G C A G T C | 1e-608 | -1401.872363 | 84.14% | 29.41% | motif file (matrix) |
| 11 | 0.925 | A G T C A G C T A G T C C G A T A T G C A C G T A C G T A G T C A G T C A G C T A G T C G A C T G A T C G A C T | 1e-587 | -1352.924466 | 85.28% | 31.46% | motif file (matrix) |
| 12 | 0.927 | C T G A A T G C A C G T A C G T A G T C A G T C A C G T | 1e-572 | -1317.191745 | 64.63% | 15.39% | motif file (matrix) |
| 13 | 0.884 | T C A G T G A C C G T A A C T G C G T A C T A G T C A G T C G A C T G A T A C G G C A T C A T G T C A G | 1e-334 | -771.196429 | 35.19% | 6.26% | motif file (matrix) |
| 14 | 0.866 | C T G A C A T G C A T G C T A G T C A G T G C A C T G A T C A G C A G T C T A G T C A G | 1e-312 | -719.180407 | 49.08% | 14.43% | motif file (matrix) |
| 15 | 0.904 | C T A G T G C A G T A C T C G A C T A G A C T G G T C A G C T A A C T G G A C T T A C G T C G A | 1e-306 | -705.474146 | 43.67% | 11.39% | motif file (matrix) |
| 16 | 0.865 | C T A G T A C G T G C A T C G A G C T A C A T G A T C G G T C A C T G A C A T G G C A T C T G A C T A G | 1e-297 | -684.450879 | 33.74% | 6.54% | motif file (matrix) |
| 17 | 0.811 | A C G T A C G T A G T C A G T C A G C T A T G C A C G T | 1e-232 | -536.239412 | 54.48% | 22.04% | motif file (matrix) |
| 18 | 0.850 | C G A T G T A C C A G T T A C G G C T A A C T G A C T G C G T A C G T A T C A G T G A C T A C G C T A G | 1e-186 | -430.581843 | 31.85% | 9.14% | motif file (matrix) |
| 19 | 0.855 | A C G T C A G T T A C G A C T G A C T G C G T A G T C A A C T G A C G T A C T G T C G A C A T G T C A G | 1e-170 | -391.903117 | 15.77% | 2.12% | motif file (matrix) |
| 20 | 0.850 | A T G C C T G A A T G C G C A T A C G T G T A C G A T C A T G C G A C T C G A T | 1e-161 | -372.106354 | 29.53% | 8.87% | motif file (matrix) |
| 21 | 0.844 | A G C T A G T C G T C A A G T C G C A T A C G T A T G C G A T C A C G T C T A G A G C T A C G T A G C T | 1e-158 | -364.180756 | 24.30% | 6.15% | motif file (matrix) |
| 22 | 0.821 | A T G C A C G T A C G T A G T C A G T C A C T G A T C G A C G T G A C T A C G T | 1e-149 | -345.380185 | 16.43% | 2.78% | motif file (matrix) |
| 23 | 0.792 | A C T G C G T A C T A G C T A G T G C A C G T A T C A G C G A T A C T G C A T G A C T G C A G T | 1e-147 | -340.247509 | 25.31% | 7.08% | motif file (matrix) |
| 24 | 0.838 | G T C A A C T G A T C G G T C A C G T A T C A G C G A T A T C G A G T C C G A T A C T G | 1e-145 | -335.132221 | 16.92% | 3.09% | motif file (matrix) |
| 25 | 0.756 | A T G C G C A T C A G T T A G C A G T C A C G T A T C G C A G T G A C T A C G T A C T G | 1e-140 | -323.433813 | 17.09% | 3.29% | motif file (matrix) |
| 26 | 0.780 | A T G C G T C A A G T C G A C T G A C T G A T C G T A C G A C T A C T G A C G T C A G T A C G T | 1e-124 | -285.772588 | 12.35% | 1.81% | motif file (matrix) |
| 27 | 0.781 | T A G C A T G C G T C A A T G C A C G T C G A T A T G C A G T C A C G T C T A G C G A T C G T A | 1e-117 | -269.441144 | 14.10% | 2.65% | motif file (matrix) |
| 28 | 0.816 | G C A T C T A G T G C A C T A G C A T G C G T A C G T A T A G C A G C T T A G C | 1e-113 | -261.713395 | 18.06% | 4.59% | motif file (matrix) |
| 29 | 0.793 | C G T A C T A G A C T G C G T A C G T A T A C G G C A T A T C G T A C G A G T C A C G T | 1e-107 | -247.617304 | 10.54% | 1.51% | motif file (matrix) |
| 30 | 0.811 | T C G A C T G A C T G A C T G A T A C G T C G A C T A G C T A G C G T A T G C A T C A G T A G C T G A C | 1e-102 | -235.746942 | 19.16% | 5.61% | motif file (matrix) |
| 31 | 0.850 | C T G A T G C A G C A T T C G A C A T G A T C G G T C A C G T A A T C G A C G T T A C G G C T A C T G A T A C G | 1e-99 | -228.804842 | 10.50% | 1.66% | motif file (matrix) |
| 32 | 0.845 | C T G A C T G A T A C G T G C A C T A G A C T G C T G A C T G A T A C G T G A C | 1e-82 | -188.894010 | 33.26% | 16.28% | motif file (matrix) |
| 33 | 0.801 | A G T C A G C T A G C T A G T C A G T C T A G C A G T C G A C T | 1e-72 | -167.701619 | 31.63% | 15.86% | motif file (matrix) |
| 34 | 0.794 | A G C T A T C G A T G C G T C A A T G C A C G T A C G T G T A C G T A C A T G C C G A T | 1e-66 | -153.253400 | 11.91% | 3.28% | motif file (matrix) |
| 35 | 0.803 | C G A T C T G A A T G C A C G T A C G T G T A C A G T C C G A T A T C G G A T C A T G C | 1e-62 | -144.878740 | 9.97% | 2.46% | motif file (matrix) |
| 36 | 0.773 | T C A G A C T G A T C G A T C G C A G T A T C G C T G A C T A G A T C G T C G A C G T A A T C G A C G T | 1e-52 | -120.803460 | 3.08% | 0.16% | motif file (matrix) |
| 37 | 0.779 | C G T A C T G A A T G C A C G T A C G T A G T C A G T C A G C T A G C T A G T C A G T C A C G T T A C G | 1e-33 | -77.031246 | 3.12% | 0.41% | motif file (matrix) |
| 38 | 0.754 | G T C A T A G C C T G A A C T G A C G T A C G T G T A C G T A C C A G T A G T C G T C A A T C G | 1e-27 | -63.097561 | 3.03% | 0.51% | motif file (matrix) |
